# CDK4/6 Inhibition Enhances the Efficacy of Standard Chemotherapy Treatment in Malignant Pleural Mesothelioma Cells

**DOI:** 10.3390/cancers14235925

**Published:** 2022-11-30

**Authors:** Rita Terenziani, Maricla Galetti, Silvia La Monica, Claudia Fumarola, Silvia Zoppi, Roberta Alfieri, Graziana Digiacomo, Andrea Cavazzoni, Delia Cavallo, Massimo Corradi, Marcello Tiseo, Pier Giorgio Petronini, Mara Bonelli

**Affiliations:** 1 Department of Medicine and Surgery, University of Parma, 43126 Parma, Italy; 2 Department of Occupational and Environmental Medicine, Epidemiology and Hygiene, INAIL-Italian Workers’ Compensation Authority, Monte Porzio Catone, 00078 Rome, Italy; 3 Center of Excellence for Toxicological Research (CERT), University of Parma, 43126 Parma, Italy; 4 Medical Oncology Unit, University Hospital of Parma, 43126 Parma, Italy

**Keywords:** malignant pleural mesothelioma, CDK4/6 inhibitors, abemaciclib, chemotherapy, senescence

## Abstract

**Simple Summary:**

Malignant pleural mesothelioma (MPM) is an aggressive disease affecting the pleura, and its incidence is increasing worldwide. Currently, the recommended systemic therapy for MPM is cisplatin/pemetrexed or immunotherapy with nivolumab and ipilimumab. Unfortunately, the prognosis remains poor, and there is an urgent need for new and effective treatments. In this study we investigated the potential antitumoral effects of combining abemaciclib with the standard chemotherapy drugs cisplatin and pemetrexed.

**Abstract:**

Background: The loss of the *CDKN2A/ARF* (cyclin-dependent kinase inhibitor 2A/alternative reading frame) gene is the most common alteration in malignant pleural mesothelioma (MPM), with an incidence of about 70%, thus representing a novel target for mesothelioma treatment. In the present study, we evaluated the antitumor potential of combining the standard chemotherapy regimen used for unresectable MPM with the CDK4/6 (cyclin-dependent kinase 4 or 6) inhibitor abemaciclib. Methods: Cell viability, cell death, senescence, and autophagy induction were evaluated in two MPM cell lines and in a primary MPM cell culture. Results: The simultaneous treatment of abemaciclib with cisplatin and pemetrexed showed a greater antiproliferative effect than chemotherapy alone, both in MPM cell lines and in primary cells. This combined treatment induced cellular senescence or autophagic cell death, depending on the cell type. More in detail, the induction of cellular senescence was related to the increased expression of p21, whereas autophagy induction was due to the impairment of the AKT/mTOR signaling. Notably, the effect of the combination was irreversible and no resumption in tumor cell proliferation was observed after drug withdrawal. Conclusion: Our results demonstrated the therapeutic potential of CDK4/6 inhibitors in combination with chemotherapy for the treatment of MPM and are consistent with the recent positive results in the MiST2 arm in abemaciclib-treated patients.

## 1. Introduction

Malignant pleural mesothelioma (MPM) is a malignant tumor of the pleura, predominantly asbestos-related and supported by a chronic inflammatory process. This rare and aggressive malignancy is characterized by poor prognosis, with a steadily increasing incidence in recent years mainly due to the latency time, estimated up to 50 years after exposure. Although processing of asbestos has been banned, at least in many Western countries, a peak of incidence is predicted in the next decade [1].

The current therapeutic actions in the first-line setting are limited to chemotherapy with cisplatin/pemetrexed (regimen that in some conditions may be improved by the addition of bevacizumab). Following the results of the CheckMate 743 study, the combination of the immune checkpoint inhibitors nivolumab and ipilimumab has been recently approved as first-line treatment in patients with unresectable MPM [2]. However, the prognosis remains poor, with a mean overall survival of about 12–14 months for patients treated with chemotherapy and around 18 months for those treated with immunotherapy.

Furthermore, there is no approved second-line treatment option and novel therapeutic strategies are urgently needed [3].

Investigating the genomic background of MPM is important for the identification of molecular targets that can be exploited for designing new therapeutic approaches in the context of precision medicine [4,5]. MPM is currently considered a heterogeneous tumor with a variety of genomic and molecular alterations, including the loss of tumor suppressor genes such as *CDKN2A/ARF* (cyclin-dependent kinase inhibitor 2A/alternative reading frame), *BAP1* (BRCA1 Associated Protein 1), and *NF2* (neurofibromatosis type 2), single-nucleotide polymorphisms (SNPs) in genes involved in DNA repair mechanisms [6], and epigenetic and non-coding RNAs mutations [5,7].

In particular, the *CDKN2A/ARF* tumor suppressor gene is frequently inactivated in MPM, with a percentage of incidence varying from 50% in the epithelioid subtype to nearly 100% in the sarcomatoid histotype [4,5]. *CDKN2A/ARF* codes for p16INK4a and its alternate reading frame p14ARF, two cell cycle proteins that negatively regulate the cell cycle progression.

p16INK4a binds to and inhibits cyclin-dependent kinase (CDK) 4 or 6, preventing their association with cyclin D1 (CycD1) and the subsequent phosphorylation of retinoblastoma (Rb) protein. By maintaining Rb in a hypo-phosphorylated state, it promotes Rb binding to E2F transcriptional factor and leads to G1 cell-cycle arrest. On the other hand, p14ARF binds to MDM2 (Mouse double minute 2 homolog) and inhibits MDM2-induced degradation of p53, enhancing p53-dependent transactivation, cell-cycle arrest, and/or apoptosis. Since the loss of *CDKN2A/ARF* occurs with high frequency in MPM patients [4,5], the use of CDK4/6 inhibitors might represent a new therapeutic option for the treatment of MPM patients.

In our previous studies, we demonstrated the efficacy of the treatment with the CDK4/6 inhibitor palbociclib alone or in combination with PI3K (Phosphoinositide 3-kinases) inhibitors in a panel of MPM cell lines [8,9], and recently these findings have been corroborated by other studies [10]. More importantly, the phase 2 clinical trial Mist2 showed encouraging results in MPM tumors, demonstrating the clinical activity of abemaciclib treatment in these patients [11].

Thus, the present work was addressed to evaluate the antitumor potential of combining the standard chemotherapeutic agents (cisplatin plus pemetrexed) used for treatment of unresectable MPM with CDK4/6 inhibitors and, in particular, with the new promising drug abemaciclib. Abemaciclib differs from other CDK inhibitors because it exerts some inhibitory effects on CDK9 in addition to targeting CDK4/6 [12]. Moreover, it is the only inhibitor in this class with activity as a single agent in breast cancer and other solid tumors [13], probably because of its peculiar characteristics. Indeed, abemaciclib has the highest affinity towards its targets [14] and a low myelotoxicity [15], and it is administered on a continuous dosing schedule, while palbociclib and ribociclib need a period of interruption [16].

Our findings demonstrate that the simultaneous treatment of abemaciclib combined with cisplatin and pemetrexed has greater antiproliferative effects than the chemotherapy in MPM cell lines. Moreover, this simultaneous treatment shows cytostatic effects in MSTO-211H and ZS-LP cells, causing senescence, while induction of autophagic cell death is observed in H28 cells. Interestingly, after drug withdrawal, tumor cell proliferation was not restored, indicating that the effects of this drug combination are irreversible.

## 2. Materials and Methods

### 2.1. Cell Culture

Human MPM cell lines MSTO-211H (biphasic histotype) and H28 (epithelioid histotype) cells were purchased by the American Type Culture Collection ATCC (Manassas, VA, USA), which authenticates the phenotypes of these cell lines on a regular basis. ZS-LP primary cell line was obtained from one patient (male, 66 years for ZS-LP) affected by mesothelioma biphasic histotype of stage T4 N0, as previously reported [8].

Cells were cultured in RPMI supplemented with 2 mM glutamine, 10% fetal bovine serum (FBS), and 100 U/mL penicillin/100 µg/mL streptomycin (Sigma Aldrich, St. Louis, MO, USA) and maintained at 37 °C in a humidified atmosphere containing 5% CO_2_.

### 2.2. Drug Treatment

Abemaciclib was provided by Selleckchem (Houston, TX, USA). Pemetrexed and cisplatin were from inpatient pharmacy of University Hospital of Parma. Abemaciclib was dissolved in sterile water, while pemetrexed and cisplatin were dissolved in 0.9% sodium chloride solution and diluted in fresh medium before use.

### 2.3. Analysis of Cell Proliferation

Cell viability/proliferation was assessed by cell counting in a Burker hemocytometer with trypan blue (Sigma Aldrich, St. Louis, MO, USA) exclusion method and by Crystal Violet (CV) or MTT assay, as previously described [17].

Cell death was assessed by Hoechst 33,342 and propidium iodide (PI) dual staining and fluorescence microscopy analysis [18] or by Annexin V-FITC/PI staining (eBioscience™ Annexin V Apoptosis Detection Kit, Invitrogen™, Waltham, MA, USA) and flow cytometry analysis; analysis of distribution of the cells in the cell cycle (performed by PI staining and flow cytometry) was described elsewhere [18].

### 2.4. Colony Formation Assay

Cells were seeded in 6-well culture plates at a density of 1.5 × 10^3^ cells/well for MSTO-211H cell lines and 2 × 10^3^ cells/well for H28 and ZS-LP cell lines. Cells were incubated at 37 °C in 5% CO_2_ incubator and the medium was changed every 3 days; at the end of the experiment, the cells were fixed with ice-cold methanol and stained with 0.1% CV (Sigma Aldrich, St. Louis, MO, USA). The unbound dye was removed by washing with water. The bound CV was solubilized with 0.2% TritonX-100 in PBS and the absorbance of the solution was measured at a wavelength of 570 nm.

### 2.5. Drug Combination Studies

To study the combined effects of cisplatin + pemetrexed with abemaciclib we used the Bliss criterion [19,20]. The Bliss criterion is expressed by the following equation: E(Bliss) = E(A) + E(B) − E(A) * E(B), where EA and EB are the percent of inhibition versus control obtained by ciplatin + pemetrexed (A) and abemaciclib (B) treatment, respectively, and the E Bliss is the theoretical dose–response curve that would be expected if the combination was exactly additive. If the combination effect (“exp” curve) is higher than the expected Bliss equation value (“Bliss” curve), the interaction is synergistic, while if the effect is lower, the interaction is antagonistic. Otherwise, the effect is additive and there is no interaction between the drugs.

The type of drug interaction was also determined by combination index (CI). CIs were calculated with Calcusyn software version 2.0 (Biosoft, Cambridge, UK) based on the method of Chou and Talalay: CI < 0.8 is considered as synergistic, 0.8 < CI < 1.2 additive and CI > 1.2 antagonistic [21].

### 2.6. Senescence Evaluation

The evaluation of senescence-associated β-galactosidase (SA-β-Gal) expression was performed using the Senescence β-Galactoside Staining kit (Cell Signaling Technology Inc., Beverly, MA, USA) as previously described [14]. The number of SA-β-Gal positive cells (blue stained) was evaluated by cell counting in four randomly chosen microscope fields (100× magnification).

### 2.7. Western Blotting

Protein extraction, solubilization, and protein analysis by Western blotting were performed as described [17]. A detailed list of the primary selected antibodies is reported in Supplementary Materials (Table S2). Horseradish peroxidase–conjugated secondary antibodies and chemiluminescence system were from Millipore (Burlington, MA, USA). Reagents for electrophoresis and blotting analysis were from BIO-RAD Laboratories (Hercules, CA, USA). The chemiluminescent signal was acquired by C-DiGit^®^. Blot Scanner and the spots were quantified by Image StudioTM Software, LI-COR Biotechnology (Lincoln, NE, USA).

### 2.8. Analysis of Autophagy

The induction of autophagy was determined using the CYTO-ID^®^ autophagy detection kit 2.0 (Enzo Life Sciences, Farmingdale, NY, USA), according to the producer’s procedures. For microscopic analysis, cells were seeded and cultured on microscopy slides to approximately 70% confluence before treatment with drugs at indicated concentrations. Untreated cells were used as negative controls. After 48 h, the cells were carefully washed and the fluorescent dyes (CYTO-ID^®^ Green Detection Reagent and Hoechst 33,342 Nuclear Stain) were applied to the slides for 30 min at 37 °C. Then, the cells were washed twice and fixed with 4% paraformaldehyde for 20 min. The sample were observed using a confocal system (Leica Stellaris 5/DMi8 platform (Leica Microsystems, Wetzlar, Germany) with LAS X software version 3.0 (Leica Microsystems, Wetzlar, Germany) with a 43/1.30 oil immersion objective. Image acquisition was carried out in the multitrack mode, namely through consecutive and independent optical pathways.

For the plate reader measurement, the CYTO-ID^®^ Green Detection Reagent and Hoechst 33,342 were added to cells growing in 96-well plates, and fluorescence measurements were recorded at λex = 480 nm and λem = 530 nm and at λex = 360 nm and λem = 460 nm, respectively, using the M200 Tecan plate reader. Increases in the green autophagy signal after normalization with blue signal indicate the accumulation of the probe within the cells arising from an increase in autophagic vesicles. Data are expressed as fold increase in the green autophagy signal versus control and are reported as autophagy induction.

### 2.9. Statistical Analysis

Statistical analyses were carried out using GraphPad Prism version 6.0 software (GraphPad Software, San Diego, CA, USA). Statistical significance of differences among data was estimated by Student’s *t*-test or by analysis of variance (one-way ANOVA) followed by Bonferroni’s post-test, and *p*-values are indicated where appropriate in the figures and in their legends. *p*-Values less than 0.05 were considered significant.

## 3. Results

### 3.1. The Association of Abemaciclib with Chemotherapy Agents Strongly Inhibited MPM Cell Proliferation

As we previously reported, MPM cell lines, as well as ZS-LP primary cells obtained from the pleural effusion of an MPM patient, displayed the molecular features associated with sensitivity to CDK4/6 inhibitors (Rb expression and p16INK4a loss) and were sensitive to palbociclib, with IC_50_ ranging from 0.1–1 μM [8]. Recently, the MiST2 trial proved the efficacy of the CDK4/6 inhibitor abemaciclib in MPM p16^INK4a^-negative patients [11].

Based on these findings, we investigated the effects of abemaciclib in combination with chemotherapy drugs currently used for the treatment of MPM patients (cisplatin plus pemetrexed) by evaluating different schedules of combination: a simultaneous treatment with abemaciclib and chemotherapy for 72 h, a sequential treatment (24 h of abemaciclib followed by chemotherapy for 48 h), and a sequential combined treatment (24 h of abemaciclib, followed by the combination of abemaciclib and chemotherapy for 48 h).

To explore the drug interaction mechanisms, with the perspective to reduce treatment toxicity, all the chemotherapy drugs were used at sub-IC_50_ concentrations (IC_50_ values for abemaciclib, cisplatin, and pemetrexed are shown in Table S1).

**Figure 1:**
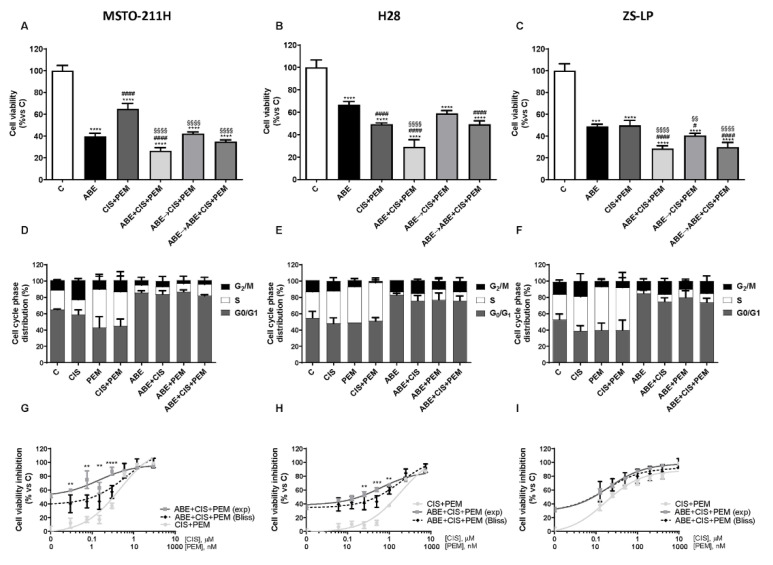
Effects of abemaciclib combined with cisplatin and pemetrexed on cell growth in MPM cell lines. (**A**) MSTO-211H cells were treated with abemaciclib alone (ABE, 0.5 µM), with the combination of cisplatin (0.3 µM) and pemetrexed (0.02 µM) (CIS + PEM), or with abemaciclib and chemotherapy following different schedules of treatment: simultaneous drug treatment for 72 h (ABE + CIS + PEM), sequential treatment with abemaciclib for 24 h followed by chemotherapy for 48 h (ABE → CIS + PEM), and sequential combined treatment (abemaciclib for 24 h followed by the combined treatment for 48 h, ABE → ABE + CIS + PEM). (**B**) H28 and (**C**) ZS-LP cells were exposed to the above-described treatments, using abemaciclib 1 µM, cisplatin 1 µM, and pemetrexed 0.05 µM. Cell viability was assessed by MTT assay and data, expressed as a percentage of cell proliferation versus control, were representative of three independent experiments. Comparison among groups was made by using analysis of variance (one-way ANOVA), followed by Bonferroni’s post-test. **** *p* < 0.0001 vs. control cells; §§ *p* < 0.01, §§§§ *p* < 0.0001 vs. cisplatin + pemetrexed-treated cells; # *p* < 0.05, #### *p* < 0.0001 vs. abemaciclib-treated cells. For the evaluation of the cell distribution in the cell cycle phases, (**D**) MSTO-211H, (**E**) H28, and (**F**) ZS-LP cells were treated with abemaciclib, cisplatin, and pemetrexed alone or simultaneously combined. After 24 h MPM cell lines were stained with PI and cell distribution in the cell-cycle phases was evaluated by flow cytometry. Data are expressed as a percentage of distribution in each cell-cycle phase and are the media of three separate experiments. The type of interaction (synergistic, additive, antagonistic) was evaluated through Bliss analysis, as described in Section 2. (**G**) MSTO-211H, (**H**) H28, and (**I**) ZS-LP cells were simultaneously treated with abemaciclib and with increasing concentrations of cisplatin and pemetrexed for 72 h. Then, cell viability was assessed by MTT assay. Data are expressed as percentage of inhibition versus control and are representative of three independent experiments. Student’s *t*-test ** *p* < 0.01, *** *p* < 0.001, **** *p* < 0.0001.

As shown in Figure 1A–C, in all cell models analyzed, the simultaneous treatment gave rise to a stronger inhibition of cell proliferation compared to abemaciclib or chemotherapy alone or to the sequential treatments. The association of chemotherapy with palbociclib produced comparable results, as shown in MSTO-211H cells (Figure S1A).

The analysis of the cell-cycle phase distribution revealed, as expected, an increase in cells in the S or G_2_/M phases after chemotherapy exposure, whereas the treatment with abemaciclib, by inhibiting the CDK4/6-Cyc D complexes, induced a cell-cycle blockade in the G_0_/G_1_ phase of the cell cycle, similarly to that observed after palbociclib treatment [8]. Interestingly, the simultaneous treatment with abemaciclib and chemotherapy promoted a cell-cycle blockade in the G_0_/G_1_ phase in all cell models analyzed (Figure 1D–F).

Comparing the sequential treatments, the one maintaining abemaciclib in the presence of chemotherapy gave the best results in term of inhibition of cell viability, indicating that maintaining CDK4/6 inhibition is required to potentiate the efficacy of chemotherapy.

Interestingly, in the simultaneous treatment, the presence of abemaciclib enhanced the efficacy of cisplatin or pemetrexed with effects comparable to those observed when both the chemotherapeutic drugs were administered together with abemaciclib (Figure S2). Similar results were observed when the cells were treated with palbociclib (Figure S1B).

Since the simultaneous combination produced the strongest inhibition of cell proliferation, we focused on this schedule. We evaluated the nature of the interaction between the treatments by using the Bliss experimental model [19,20] and demonstrated a synergistic inhibitory effect on cell proliferation in MSTO and H28 cells and an additive inhibitory effect in ZS-LP cells (Figure 1G–I). Moreover, we confirmed this type of interaction also by calculating the combination index (CI), as shown in Figure S3.

To obtain insight into the molecular mechanisms underlying the inhibition of cell proliferation promoted by the simultaneous treatment with chemotherapy and abemaciclib, we evaluated the expression level and the activation status of proteins involved in the regulation of the cell cycle and in signal transduction.

**Figure 2:**
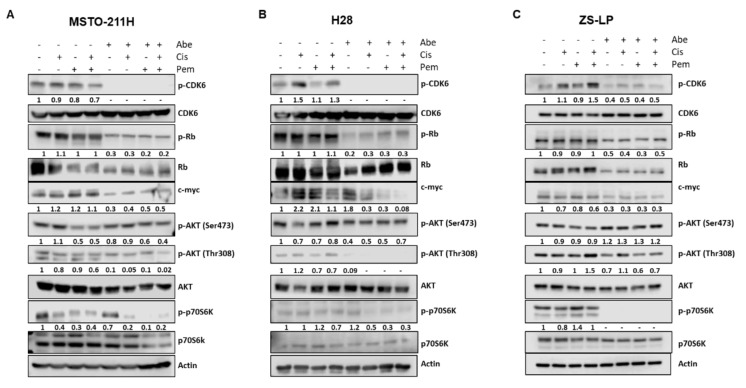
Western blotting analysis of the expression level or activation status of proteins involved in the regulation of the cell cycle and in signal transduction pathways. (**A**) MSTO-211H cells were simultaneously treated with abemaciclib (0.5 µM), cisplatin (0.3 µM), and pemetrexed (0.02 µM) for 24 h. (**B**) H28 cells were treated with abemaciclib (1 µM), cisplatin (1 µM), and pemetrexed (0.05 µM) for 24 h. (**C**) ZS-LP cells were treated with abemaciclib (1 µM), cisplatin (1 µM), and pemetrexed (0.05 µM) for 24 h. At the end of the treatments, the expression levels of proteins involved in cell cycle regulation and signal transduction were analyzed by Western blotting. The immunoreactive spots were quantified by densitometric analysis; the results are represented by the ratio between phosphorylated and total protein levels or between the total protein and actin levels. The dash (-) represents a non-detectable signal. Data are representative of two independent experiments. Figure S5: Whole Western blots of Figure 2.

As shown in Figure 2, the treatment with abemaciclib, alone or combined with chemotherapy, reduced CDK6 phosphorylation at Y24 in all cell lines analyzed. Considering that Y24-phosphorylation is inhibitory, this is a peculiar result, emerged also in other cell models [22,23]. Nevertheless, abemaciclib treatment resulted in the inhibition of CDK4/6-cyclin D complex activity.

Indeed, Rb phosphorylation was significantly downregulated and, as previously reported for palbociclib, the total levels of Rb were also reduced after treatment with abemaciclib. Because of Rb hypophosphorylation, the transcription factor E2F was inactivated and the expression of myc, one of its target genes, was markedly reduced. This effect was more evident when the cells were exposed to the simultaneous treatment with abemaciclib and cisplatin plus pemetrexed.

Interestingly, the treatment with abemaciclib, alone or combined with chemotherapy, reduced AKT activation and downregulated the downstream mTOR signaling, as indicated by the reduced phosphorylation of p70S6K.

**Figure 3:**
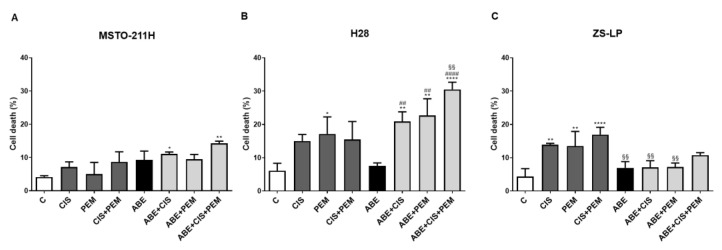
Effects of abemaciclib in association with cisplatin and pemetrexed on cell death in MPM cell lines. (**A**) MSTO-211H cells were treated with abemaciclib (0.5 µM), cisplatin (0.3 µM), and pemetrexed (0.02 µM), or with the simultaneous combination of abemaciclib and chemotherapy for 72 h. (**B**) H28, and (**C**) ZS-LP cells were exposed to the above-described treatments, using abemaciclib 1 µM, cisplatin 1 µM, and pemetrexed 0.05 µM. The percentage of dead cells stained with Hoechst 33,342 and Propidium Iodide is shown. Data are expressed as a percentage of cell death and are the medians of three separate experiments. Comparison among groups was made by using analysis of variance (one-way ANOVA), followed by Bonferroni’s post-test. * *p* < 0.05, ** *p* < 0.01, **** *p* < 0.0001 vs. control cells; §§ *p* < 0.01, vs. cisplatin + pemetrexed-treated cells; ## *p* < 0.01, #### *p* < 0.0001 vs. abemaciclib-treated cells.

We then explored whether the greater inhibition of cell proliferation observed in MPM cells treated with abemaciclib and chemotherapy was associated with the induction of cell death. As shown in Figure 3, in the MSTO-211H and ZS-LP cell lines, the simultaneous treatment with abemaciclib and chemotherapy gave rise only to a slight increase (ranging from 10 to 15%) in the percentage of cell death, thus suggesting the predominance of the cytostatic effect of abemaciclib (Figure 3A–C).

On the other hand, in H28 cells, the cytotoxic effect exerted by chemotherapy was further increased when the cells were simultaneously treated with abemaciclib and reached the highest level (more than 30%) in the presence of both cisplatin and pemetrexed (Figure 3B). The fluorescence microscopy analysis of Hoechst 3332/PI-stained cells suggested that the triple drug treatment induced cell death by non-apoptotic mechanisms. This result was confirmed by flow cytometry analysis, which showed a small increase in the percentage of Annexin V-FITC positive, PI negative cells in triple drug-treated cells in comparison with control cells (Figure S4).

### 3.2. The Combined Treatment with Abemaciclib and Chemotherapy Induced Cellular Senescence or Autophagy Depending on the Cell Model

It is well documented that the exposure to CDK4/6 inhibitors induces cellular senescence in a variety of cancer cell models and the acquisition of a senescent phenotype has been described also in MPM cell lines [8,10].

**Figure 4:**
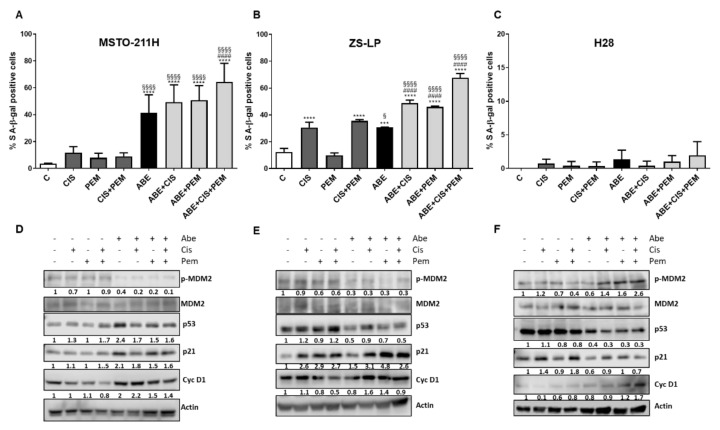
Effects of abemaciclib in association with cisplatin and pemetrexed on senescence induction in MPM cell lines. (**A**) MSTO-211H cells were treated with abemaciclib (0.5 µM), cisplatin (0.3 µM), and pemetrexed (0.02 µM), or with the simultaneous combination of abemaciclib and chemotherapy for 72 h. (**B**) ZS-LP and (**C**) H28 cells were exposed to the above-described treatments, using abemaciclib 1 µM, cisplatin 1 µM, and pemetrexed 0.05 µM. Data are expressed as a percentage of senescent cells positive for SA-β-Gal expression and are the media of three separate experiments. Comparison among groups was made by using analysis of variance (one-way ANOVA), followed by Bonferroni’s post-test. *** *p* < 0.001, **** *p* < 0.0001 vs. control cells; § *p* < 0.05, §§§§ *p* < 0.0001 vs. cisplatin + pemetrexed-treated cells; #### *p* < 0.0001 vs. abemaciclib-treated cells. (**D**) MSTO-211H, (**E**) ZS-LP, and (**F**) H28 cells were treated as described above and Western blotting analysis of proteins involved in cell cycle regulation and senescence induction was performed. The immunoreactive spots were quantified by densitometric analysis; the results are represented by the ratio between phosphorylated and total protein levels or between the total protein and actin levels. The dash (-) represents a non-detectable signal. Data are representative of two independent experiments. Figure S6: Whole Western blots of Figure 4.

Therefore, we evaluated the impact of the simultaneous drug treatment on senescence induction in MSTO-211H, H28, and ZS-LP MPM cells. As shown in Figure 4, a significant increase in senescent cells was induced by abemaciclib treatment with a percentage of senescent cells ranging around 40% in MSTO-211H cells and 30% in ZS-LP. The percentage of senescent cells was further increased after abemaciclib/chemotherapy simultaneous treatment, with stronger senescence induction after abemaciclib/cisplatin/pemetrexed treatment (70% of senescent cells in MSTO-211H and 60% in ZS-LP cells). Interestingly, in ZS-LP cells a significant induction of cellular senescence was also observed in cells treated with cisplatin (alone or in association with pemetrexed). By contrast, no senescence induction was observed in H28 cells, either after abemaciclib treatment alone or after the combined treatment with abemaciclib and chemotherapy, differently to what we previously observed with palbociclib treatment (not shown and [8]).

Then, the expression level of proteins involved in cell-cycle control and senescence induction was evaluated in MSTO-211H and ZS-LP cells (Figure 4D–E). In both the cell lines, abemaciclib alone and its combination with chemotherapy caused a strong decrease in MDM2 phosphorylation, thus inducing the accumulation of the cell-cycle negative regulators p53 and/or p21 and driving the cells into the senescence process.

In contrast with MSTO-211H and ZS-LP cells, H28 cells did not show any sign of cellular senescence and neither p53 nor p21 expression levels were induced by abemaciclib alone or in combination with chemotherapy (Figure 4C–F). However, after these drug treatments, H28 cells displayed peculiar morphology changes, such as increased cell size and formation of several cytoplasmic vacuoles.

**Figure 5:**
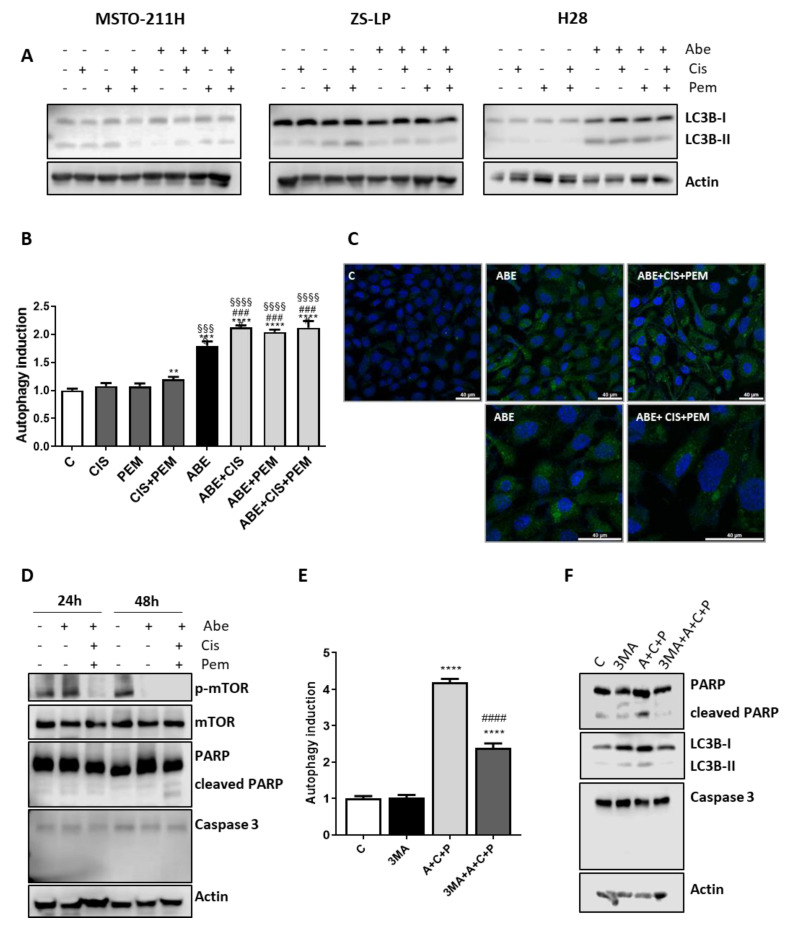
Effects of abemaciclib in association with cisplatin and pemetrexed on autophagy induction in MPM cell lines. (**A**) MSTO-211H cells were treated with abemaciclib (0.5 µM), cisplatin (0.3 µM), and pemetrexed (0.02 µM), or with the simultaneous combination of abemaciclib and chemotherapy for 72 h. ZS-LP and H28 cells were exposed to the above-described treatments, using abemaciclib 1 µM, cisplatin 1 µM, and pemetrexed 0.05 µM. Western blotting analysis of proteins involved in autophagy induction. Data are representative of two independent experiments. (**B**) H28 cells were treated as described above, and after 48 h autophagy was assessed by the CYTO-IDR Autophagy detection Kit 2.0. Data are expressed as fold increase versus control (autophagy induction) and are the medians of two separate experiments. Comparison among groups was made by using analysis of variance (one-way ANOVA), followed by Bonferroni’s post-test. ** *p* < 0.01, *** *p* < 0.001, **** *p* < 0.0001 vs. control cells; §§§ *p* < 0.001, §§§§ *p* < 0.0001 vs. cisplatin + pemetrexed-treated cells; ### *p* < 0.001 vs. abemaciclib-treated cells. (**C**) Representative images by confocal microscopy of H28 cells, treated as described above, stained with CYTO-IDR Autophagy detection Kit 2.0. (**D**) H28 cells were treated as described above, then cellular proteins were extracted and analyzed by Western blotting using the indicated antibodies. Data are representative of two independent experiments. (**E**) H28 cells were treated with the simultaneous combination of abemaciclib (1 µM), cisplatin (1 µM), and pemetrexed (0.05 µM), with or without the autophagic inhibitor 3-metyl adenine (2.5 mM). After 48 h, autophagy was assessed by the CYTO-IDR Autophagy detection Kit 2.0. Data are expressed as fold increase versus control (autophagy induction) and are the media of two separate experiments. Comparison among groups was made by using analysis of variance (one-way ANOVA), followed by Bonferroni’s post-test **** *p* < 0.0001 vs. control cells; #### *p* < 0.0001 vs. abemaciclib + cisplatin + pemetrexed-treated cells. (**F**) H28 cells were treated as described in (**E**), then cells were lysed and cell protein extracts were analyzed by Western blotting for the indicated proteins. Data are representative of two independent experiments. Figure S7: Whole Western blots of Figure 5.

Interestingly, the activation of lipidated microtubule-associated protein light-chain 3 (LC3B), considered as a marker of autophagy, was observed in H28 cells treated with abemaciclib alone or in combination with chemotherapy (Figure 5A), whereas no significant changes in the level of LC3B were observed in MSTO-211H and ZS-LP cells.

The induction of autophagy in H28 cell line was confirmed using the CYTO-ID^®^ Autophagy Detection Kit, which provides a selective marker of autolysosomes and earlier autophagic compartments. As shown in Figure 5B,C, abemaciclib treatment clearly augmented the number of autophagic cells, and a small but significant further increase was observed after the combined treatment with chemotherapy. The increase in autophagy was related to the decreased activation of mTOR, already detectable after 24 h of combined treatment (Figure 5D). After 48 h, the decrease in mTOR activity was evident also in cells treated with abemaciclib alone. The autophagy process was associated with poly (ADP-ribose) polymerase (PARP) cleavage, whereas we failed to detect caspase activation. A pattern of cell death characterized by PARP-1 cleavage without caspase activation has been recently described by Hino and co-workers in lung cancer cell lines exposed to abemaciclib [24].

To give strength to these results, we investigated the effect of 3-methyladenine (3MA), a class III phosphatidylinositol 3-kinase (PI3K)-blocking autophagy inhibitor [25], in association with the simultaneous treatment of abemaciclib and chemotherapy. As shown in Figure 5E,F, 3MA significantly hampered the induction of autophagy, reducing both the PARP-1 cleavage and the activation of LC3B. These results confirmed that in H28 cells the simultaneous drug treatment induces the autophagic process.

### 3.3. The AntiProliferative Effects of Abemaciclib Combined with Chemotherapy Were Maintained after Drug Removal

**Figure 6:**
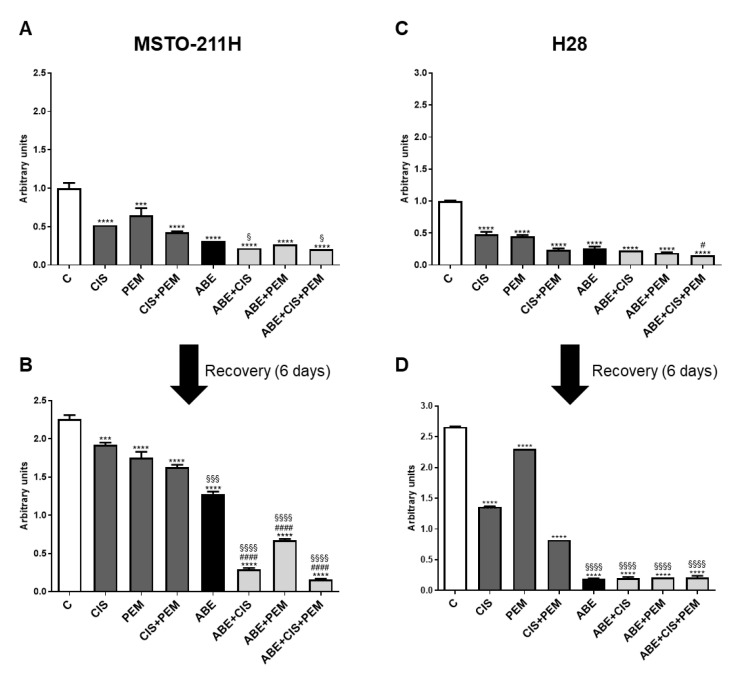
Effects of removal of abemaciclib, cisplatin, and pemetrexed on cell proliferation in MSTO-211H and H28 cells. (**A**,**B**) MSTO-211H cells were treated with abemaciclib (0.5 µM), cisplatin (0.3 µM), and pemetrexed (0.02 µM), or with the simultaneous combination of abemaciclib and chemotherapy for 6 days. (**C**,**D**) H28 cells were exposed to the above-described treatments, using abemaciclib 1 µM, cisplatin 1 µM, and pemetrexed 0.05 µM. After drug treatment, cells were cultured for additional 6 days in drug-free medium (recovery time). Cell proliferation was evaluated by crystal violet staining. Data are the medians of three separate experiments. Comparison among groups was made by using analysis of variance (one-way ANOVA), followed by Bonferroni’s post-test. *** *p* < 0.001, **** *p* < 0.0001 vs. control cells; § *p* < 0.05, §§§ *p* < 0.001, §§§§ *p* < 0.0001 vs. cisplatin + pemetrexed-treated cells; # *p* < 0.05, #### *p* < 0.0001 vs. abemaciclib-treated cells.

Finally, to assess whether the effects of the simultaneous treatment of abemaciclib and chemotherapy on cell proliferation were reversible, we performed long-term colony formation experiments. MPM cells were treated with the drugs alone or in combination for 6 days; then, the medium was replaced with drug-free medium and the cells were cultured for additional 6 days (Figure 6). After the recovery period, the colony formation capability was evaluated, demonstrating that the cells treated with cisplatin and pemetrexed, alone or in combination, restored their proliferation after drug withdrawal.

Interestingly, the combination of abemaciclib with chemotherapy yielded irreversible effects on cell proliferation, even if to different extents depending on the cell model. Indeed, in MSTO-211H cells, abemaciclib alone failed to demonstrate an irreversible effect, whereas the combination of abemaciclib with chemotherapy significantly maintained the cell growth inhibition even after drug removal. The inhibitory effect was markedly increased when abemaciclib was combined both with cisplatin and pemetrexed, indicating that this treatment more efficaciously determined an irreversible inhibition of cell proliferation (Figure 6A,B).

In H28 cells, abemaciclib alone was sufficient to inhibit cell proliferation irreversibly, an effect that was maintained even in combination with chemotherapy (Figure 6C,D).

## 4. Discussion

In the last few years, relevant progresses have been made in the treatment of MPM; indeed, the results of the CheckMate 743 study brought to the introduction of immunotherapy, based on the combination of nivolumab and ipilimumab, as first-line treatment in patients with unresectable MPM [2]. However, the prognosis for MPM patients remains poor with a mean overall survival around 18 months.

In addition, the lack of oncogene-driver mutations has limited the development of targeted therapies for this type of tumor. Indeed, a number of genetic analyses revealed that MPM is characterized mainly by the loss of function of oncosuppressor genes.

The high frequency of loss of function of the *CDKN2A/ARF* gene prompted us and other groups to investigate the target approach in MPM cell lines using CDK4/6 inhibitors, and recently the efficacy of the CDK4/6 inhibitor abemaciclib has been proven in the MiST2 trial in MPM patients harboring p16INK4a loss [11].

These successful findings open a new possibility of treatment for MPM patients. However, CDK4/6 inhibitors, currently approved for the treatment of estrogen receptor- positive breast cancer, displayed mainly a cytostatic effect when administered alone, whereas combination therapies demonstrated an enhanced efficacy in different tumor types [26,27].

Focusing on the combination of CDK4/6 inhibitors with chemotherapy, some controversial data have emerged in recent years; indeed, the association between CDK4/6 inhibitors and diverse chemotherapeutic agents frequently produced an antagonistic effect due to the reduced sensitivity to chemotherapy of cell cycle-arrested cells [28,29]. However, the timing, the drug concentration and the sequence of drug exposure might play a critical role in drug activity, as recently demonstrated in different tumor types. In particular, the sequential combination of CDK4/6 inhibitors and paclitaxel-based chemotherapy produced antiproliferative and pro-apoptotic effects in TNBC (triple-negative breast cancer) cell lines associated with an impairment of glucose metabolism [30], and recently, the efficacy of the simultaneous combination of gemcitabine/cisplatin-based chemotherapy and CDK4/6 inhibitors has been demonstrated also in biliary tract cancers [31].

In the present study, we demonstrated the efficacy of the simultaneous combination of CDK4/6 inhibitors with cisplatin and pemetrexed. In particular, our findings suggest that the CDK4/6 inhibitor abemaciclib may be considered for the treatment of MPM selected patients as a valuable tool to improve the efficacy of chemotherapy, which remains one of the standards of care for this malignancy.

Indeed, the simultaneous treatment of different MPM cell lines (two commercial and one obtained from pleural effusion of a MPM patient) with abemaciclib and the combination cisplatin/pemetrexed showed a synergistic or additive inhibition of cell proliferation, depending on the cell model.

It is worth noting that a limitation of the combined therapies is the increased risk of toxicity; taking into account this aspect, we used the chemotherapy drugs at sub-IC_50_ concentrations, demonstrating the ability of abemaciclib to improve the efficacy of chemotherapy even when administered at the lowest feasible doses. Interestingly, enhanced antitumor effects were also obtained when abemaciclib was combined with each chemotherapy agents alone, suggesting the potential of these dual-drug combinations in the light of reduced toxicity.

The inhibition of cell proliferation observed with abemaciclib combined with cisplatin and/or pemetrexed was associated with the downregulation of the AKT/mTOR signaling. In contrast, we previously reported that palbociclib induced a strong upregulation of this pathway in the same MPM cell models [8]. Of note, the opposite effects of abemaciclib and palbociclib on the AKT/mTOR signaling pathway have been previously shown in other cell models [23,27,32]. These discrepancies can be explained considering that abemaciclib may exert modulatory activities that are distinct from those of the other CDK4/6 inhibitors. In fact, it has been shown to inhibit various kinases, including CDK9 and non-CDK targets, such as DYRK2 (Dual Specificity Tyrosine Phosphorylation Regulated Kinase 2), PIM1, HIPK2 (Homeodomain Interacting Protein Kinase 2), and CK2 (casein kinase II) [12]. Therefore, we can speculate that these additional inhibitory activities may have an impact on the regulation of the AKT/mTOR pathway, as demonstrated in other studies [33,34].

The combination of abemaciclib with chemotherapy-induced cell senescence or death, depending on the cell model. In particular, in MSTO-211H and ZS-LP cells, the simultaneous triple drug treatment significantly increased the percentage of senescent cells in comparison to abemaciclib alone or abemaciclib in association with either cisplatin or pemetrexed. Mechanistically, the induction of senescence was sustained by the p53/p21 axis. It is well documented that p21 plays a relevant role in the induction of cellular senescence in both normal and tumor cells through p53-dependent or -independent mechanisms, as we observed in MSTO-211H and ZS-LP cells, respectively [35]. In fact, the treatment with abemaciclib produced a decrease in MDM2 activity in both cell models (probably due to the reduced activation of AKT). This resulted in the stabilization of both p53 and p21 proteins in MSTO-211H cells, whereas in ZS-LP cells p21 accumulation occurred in a p53-independent manner; indeed, MDM2 can directly control p21 stability, and MDM2 downregulation can lead to increased p21 levels independently of p53 activity [36].

It is worth noting that in ZS-LP cells, cisplatin alone induced an accumulation of senescent cells, which was further increased by the combination with abemaciclib. This effect mediated by chemotherapy has already been described in different cancer types, such as ovarian and melanoma, and could be attributed to the increase in p21 expression [37,38].

Differently, in H28 cells, we observed an increased cell death after the simultaneous treatment with abemaciclib and cisplatin or pemetrexed alone compared to single agent treatments; this effect was further enhanced after triple-drug combination treatment. It is worth of note that in this cell model the treatment with abemaciclib alone or combined with chemotherapy induced morphological changes, such as an enlarged cell shape and accumulation of many large cytoplasmic vacuoles. A comparable phenomenon was described in a different tumor cell model: in Rb-negative breast cancer cell lines, abemaciclib induced a cytotoxic effect associated with a multivacuolar phenotype, showing an off-target effect [39]. Recently, in A549 lung cancer cells, an atypical cell death induced by abemaciclib treatment and characterized by the accumulation of autophagosomes has been described, probably derived from lysosomes that expanded after acidification [24]. In the H28 cell line, the morphological changes induced by abemaciclib treatment alone or combined with chemotherapy, together with the increase in LC3B-II levels and the autolysosomes staining, indicate the induction of autophagy. It has been recently demonstrated that the Cyc D/CDK4/6 complexes regulate both the activation and the cytoplasmatic localization of mTORC1 [40,41]. Since mTOR plays a key role in controlling autophagy, we can infer that in H28 cells, the inhibition of the AKT/mTOR/p70S6K pathway is involved in the activation of autophagy mediated by the combined treatment with abemaciclib and chemotherapy.

The specific role of autophagy in cancer cells remains controversial and highly context-dependent [42]; indeed the autophagic process has been described as a mechanism of resistance to CDK4/6 inhibitors [43] as well as an inducer of cell death [44,45]. In our study, the induction of autophagy by the combined treatments is associated with increased non-apoptotic cell death in H28 cells and therefore ameliorates the drug efficacy.

One relevant aspect of our study is the demonstration that the growth inhibitory effects observed in MPM cells treated with the simultaneous combination of abemaciclib and chemotherapy are irreversible and are maintained even after drug withdrawal. In contrast, the cells treated with chemotherapy alone reacquired the proliferative capability after drug removal. Once again, the behavior of the different MPM cell lines showed some peculiar aspects: indeed, in H28 cells, the treatment with abemaciclib alone resulted in an irreversible inhibition of cell proliferation, whereas MSTO-211H cells reacquired the proliferation capability after abemaciclib removal, even if to a lesser extent as compared to chemotherapy agents. The translation of this result in the clinical setting reveals a potential role of abemaciclib in preventing the acquisition of resistance to chemotherapy agents, a phenomenon that inevitably occurs in MPM patients leading to therapy failure.

## 5. Conclusions

For patients with unresectable MPM, chemotherapy or immunotherapy remain the standard of care as first-line treatment. Nevertheless, the prognosis remains poor and novel therapeutic strategies are urgently needed. Our preclinical data strongly support the ongoing clinical evaluation of abemaciclib in p16^INK4a^-negative patients, in particular suggesting its combination with the standard chemotherapy regimen based on cisplatin plus pemetrexed. Furthermore, considering the irreversible antitumor effects of the drug combination, we might hypothesize that the maintenance of abemaciclib after the combined treatment could be a promising therapeutic option for MPM patients.

## Data Availability

The data presented in this study are available on request from the corresponding author.

## Supplementary Materials

The following supporting information can be downloaded at: https://www.mdpi.com/article/10.3390/cancers14235925/s1, Table S1. MPM cell sensitivity to abemaciclib, cisplatin and pemetrexed. Table S2. List of the antibodies used in the paper. Figure S1: Effects of palbociclib combined with cisplatin and pemetrexed on cell viability in MSTO-211H cells; Figure S2: Effects of abemaciclib combined with cisplatin and pemetrexed on cell viability in MPM cell lines; Figure S3: Effects of abemaciclib combined with cisplatin and pemetrexed on colony formation in MSTO-211H cells; Figure S4: Analysis of cell death by Annexin V/PI staining assay in H28 cells; Figure S5: Whole Western blots of Figure 2; Figure S6: Whole Western blots of Figure 4; Figure S7: Whole Western blots of Figure 5.
