# Supplementary material for: CDK4/6 Inhibition Enhances the Efficacy of Standard Chemotherapy Treatment in Malignant Pleural Mesothelioma Cells
Source: Cancers (Basel). 2022 Nov 30;14(23):5925. doi: 10.3390/cancers14235925 (PMC9739278; doi:10.3390/cancers14235925)
Supplement: Supplementary file 1 [file cancers-14-05925-s001.zip › cancers-1876167-supplementary.pdf]

cancers

MDPI

Supplementary Material: CDK4/6 Inhibition Enhances the Efficacy of Standard Chemotherapy Treatment in Malignant Pleural Mesothelioma Cells

Rita Terenziani, Maricla Galetti*, Silvia La Monica*, Claudia Fumarola, Silvia Zoppi, Roberta Alfieri, Graziana Digiacomo, Andrea Cavazzoni, Delia Cavallo, Massimo Corradi, Marcello Tiseo, Pier Giorgio Petronini and Mara Bonelli

Table S1. MPM cell sensitivity to abemaciclib, **cisplatin and pemetrexed.** MSTO-211H, H28 and ZS-LP cells were treated with increasing concentrations of abemaciclib, cisplatin, and pemetrexed for 72 h. Cell proliferation was assessed by MTT and the IC50 values were calculated using GraphPad Prism 6.00 software. Data are the mean value of three independent measurements.

Cell line	Abemaciclib (µM)	Cisplatin (µM)	Pemetrexed (nM)
**MSTO-211H**	0.5	0.4	30
**H28**	1.2	2.5	60
**ZS-LP**	1	2	50

Table S2. List of the antibodies used in the paper.

ANTIBODY	COMPANY	PRODUCT NUMBER
p-Rb (Ser780)	CST (Danvers, MA, USA)	#9307
Rb (4H1)	CST (Danvers, MA, USA)	#9309
c-Myc	CST (Danvers, MA, USA)	#9402
Cyclin D1	CST (Danvers, MA, USA)	#2922
p-MDM2 (Ser166)	CST (Danvers, MA, USA)	#3521
MDM2 (D1V2Z)	CST (Danvers, MA, USA)	#86934
p21 Waf1/Cip1 (12D1)	CST (Danvers, MA, USA)	#2947
p-CDK6 (Tyr24)	Santa Cruz Biothecnology (Dallas, Texas, USA)	sc-293097
CDK6 (DCS83)	CST (Danvers, MA, USA)	#3136
p-AKT (Ser473)	CST (Danvers, MA, USA)	#9271
p-AKT (Thr308) (244F9)	CST (Danvers, MA, USA)	#4056
AKT	CST (Danvers, MA, USA)	#9272
p-p70 S6 Kinase (Thr421/Ser424)	CST (Danvers, MA, USA)	#9204
p70 S6 Kinase	CST (Danvers, MA, USA)	#9202
p-mTOR (Ser2448)	CST (Danvers, MA, USA)	#2971
mTOR	CST (Danvers, MA, USA)	#2972
Phospho-p 44/42 MAPK (Erk1/2) (Thr202/Tyr204) (D13.14.4E)	CST (Danvers, MA, USA)	#4370
p44/42 MAPK (Erk1/2)	CST (Danvers, MA, USA)	#4695
LC3B (D11)XP	CST (Danvers, MA, USA)	#3868
Caspase-3	CST (Danvers, MA, USA)	#9662
PARP (46D11)	CST (Danvers, MA, USA)	#9532
p-53 (DO-1)	Santa Cruz Biotecnology	sc-126
-actin (15G5A11/E2)	(Massachusetts, USA)	15G5A11/E2

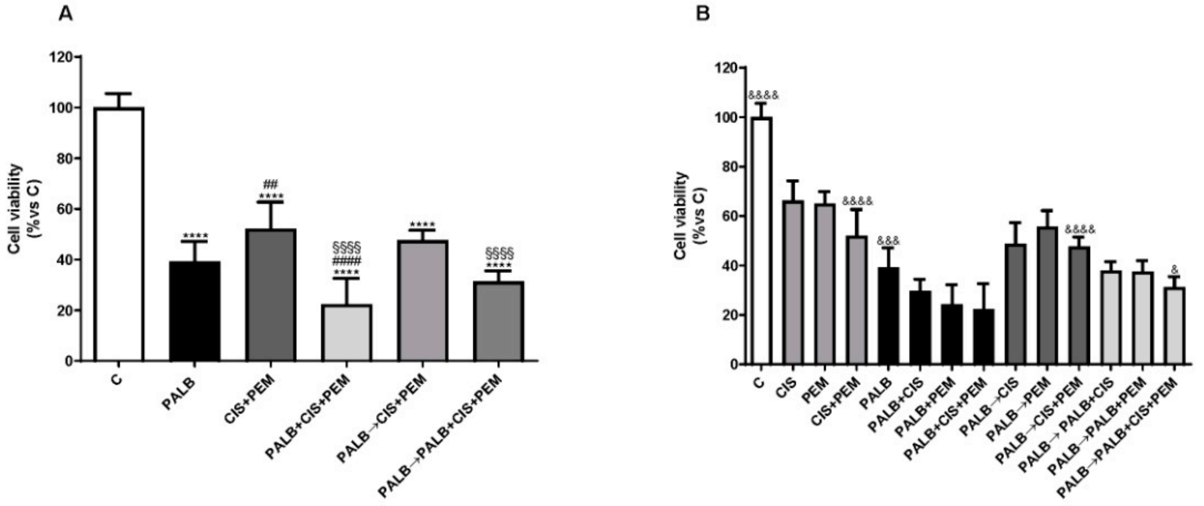

Figure S1. Effects of palbociclib combined with cisplatin and pemetrexed on cell viability in MSTO-211H cells. (A) MSTO-211H cells were treated with palbociclib alone (0.5 μM), with the combination of cisplatin (0.3 μM) and pemetrexed (0.02 μM), or with palbociclib and chemotherapy following different schedules of treatment: simultaneous drug treatment for 72 h, sequential treatment with palbociclib for 24 h followed by chemotherapy for 48 h and sequential combined treatment (palbociclib for 24 h followed by the combined treatment for 48 h). ****p<0.0001 vs. C; §§§§p<0.0001 vs CIS+PEM; ##p<0.01, ####p<0.0001 vs. abemaciclib. (B) Evaluation of the contribution of each drug combination on the inhibition of MSTO-211H cell viability: cells were exposed to the schedules described in "A" and the effect of each drug combination was evaluated in term of inhibition of cell viability. Cell viability was assessed by MTT and data, expressed as the percentage of cell viability versus control, were representative of three independent experiments. Comparison among groups was made by using analysis of variance (one-way ANOVA), followed by Bonferroni's post-test. &p<0.05, &&&p<0.001 &&&&p<0.0001 vs. PALB+CIS+PEM.

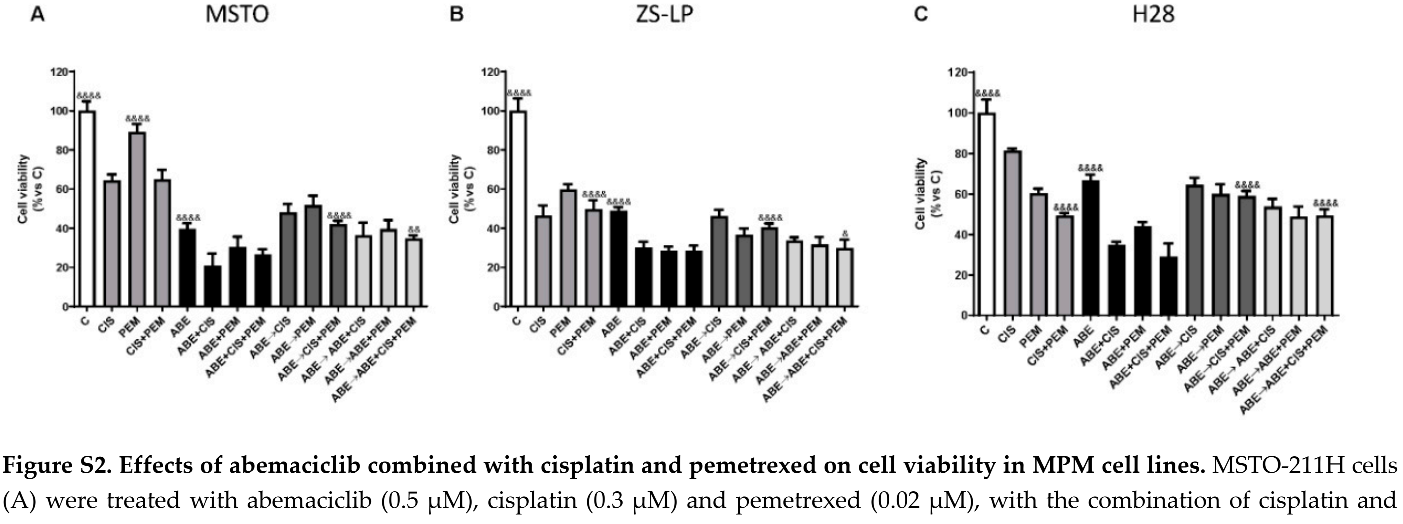

Figure S2. Effects of abemaciclib combined with cisplatin and pemetrexed on cell viability in MPM cell lines. MSTO-211H cells (A) were treated with abemaciclib (0.5 μM), cisplatin (0.3 μM) and pemetrexed (0.02 μM), with the combination of cisplatin and pemetrexed, or with abemaciclib and chemotherapy following different schedules of treatment: simultaneous drug treatment for 72 h, sequential treatment with abemaciclib for 24 h followed by chemotherapy for 48 h and sequential combined treatment (abemaciclib for 24 h followed by the combined treatment for 48 h). The effect of each drug combination was evaluated in term of inhibition of cell viability and assessed by MTT. (B) H28 and (C) ZS-LP cells were exposed to the above-described treatments, using abemaciclib 1 μM, cisplatin 1 μM and pemetrexed 0.05 μM. Cell proliferation was assessed by MTT assay and data, expressed as the percentage of cell viability versus control, were representative of three independent experiments. Comparison among groups was made by using analysis of variance (one-way ANOVA), followed by Bonferroni's post-test. &p<0.05, &&p<0.01, &&&&p<0.0001 vs. ABE+CIS+PEM.

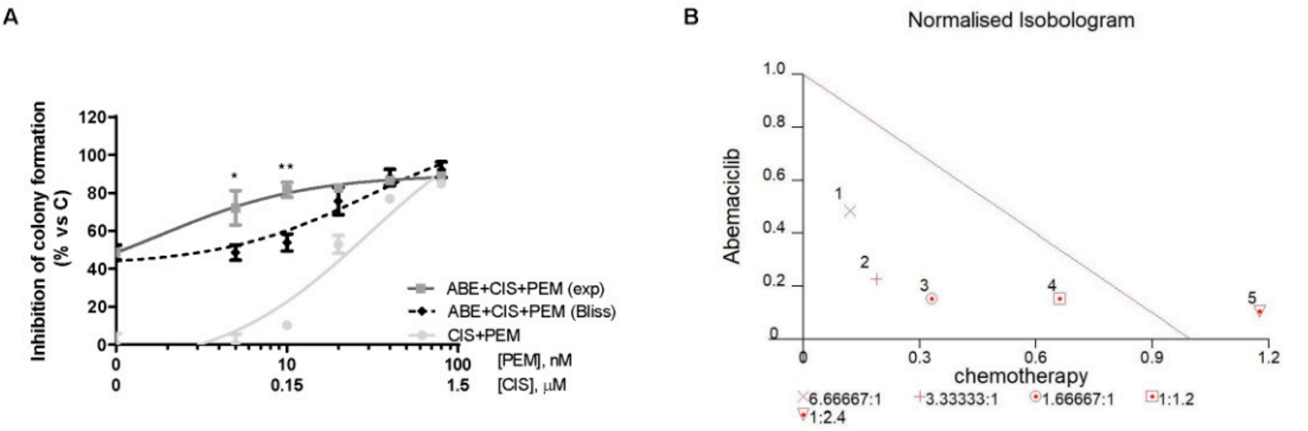

Figure S3. Effects of abemaciclib combined with cisplatin and pemetrexed on colony formation in MSTO-211H cells. MSTO-211H cells were simultaneously treated with abemaciclib (0.5 μM) and with increased concentrations of cisplatin and pemetrexed for 72 h. The effect of the drugs was assessed by crystal violet assay. The type of interaction (synergistic, additive or antagonistic) was evaluated through Bliss interaction model (A) and combination indexes were calculated with Calcusyn software (B), as described in Materials and Methods section. Data are representative of three independent experiments. Student's t test *p<0.05, **p<0.01 vs. Bliss.

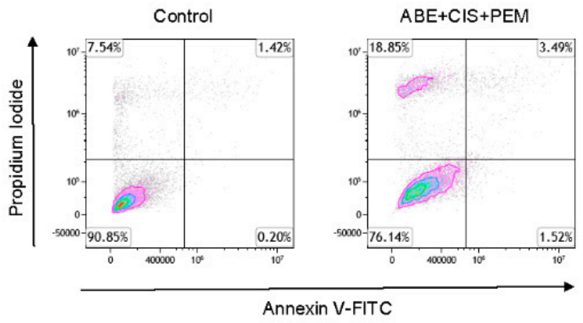

Figure S4. Analysis of cell death by Annexin V/PI staining assay in H28 cells

H28 cells were treated with the simultaneous combination of abemaciclib (1 μM), cisplatin (1 μM), and pemetrexed (0.05 μM). After 24 h the cells were analyzed for the induction of cell death by apoptosis using an Annexin V Apoptosis Detection Kit. Representative images of two independent experiments are shown.

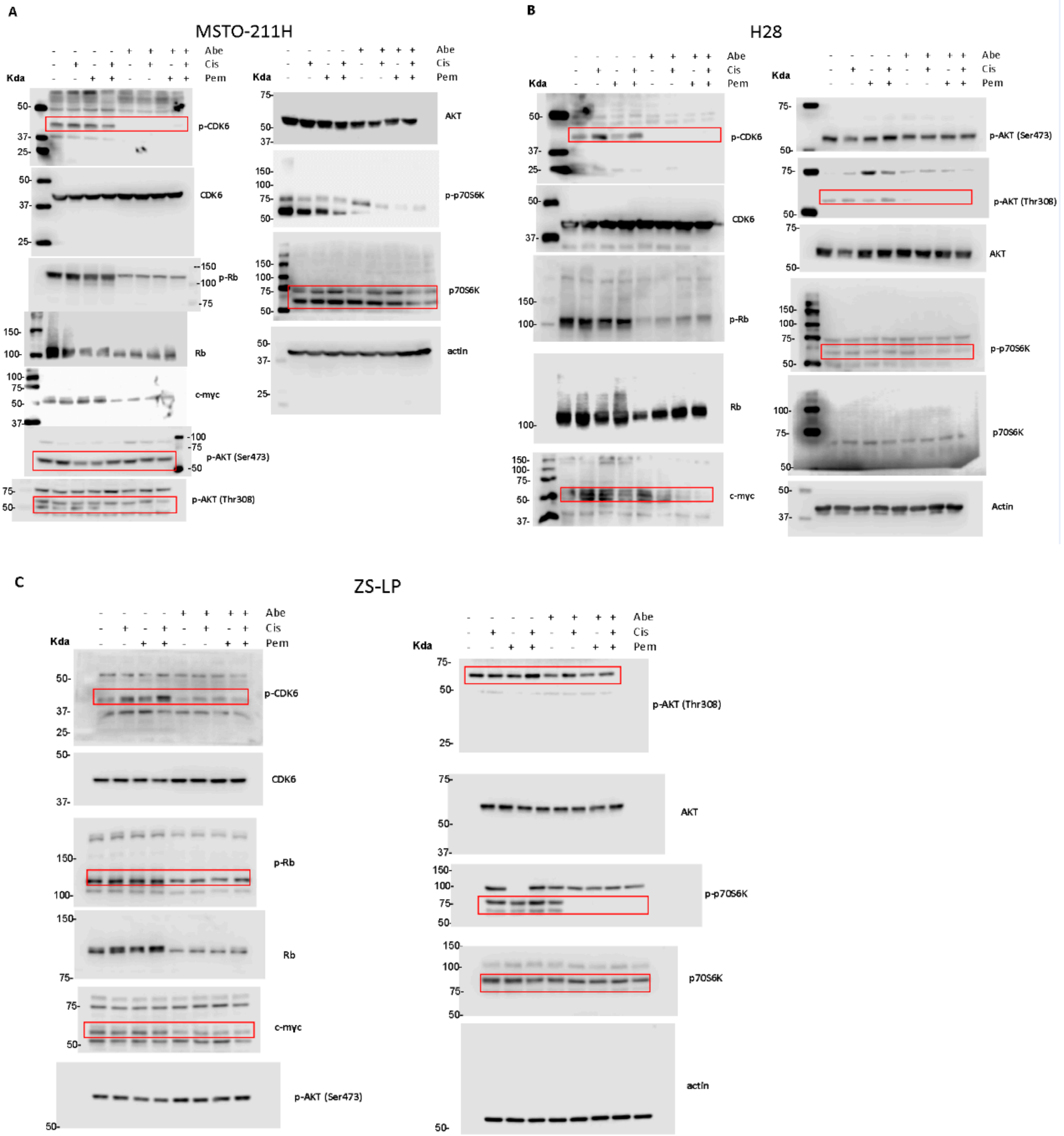

Figure S5: Full original blots used for Figure 2A, 2B e 2C. Each blot membrane was cut based on the standard band positions and then incubated with the corresponding antibodies.

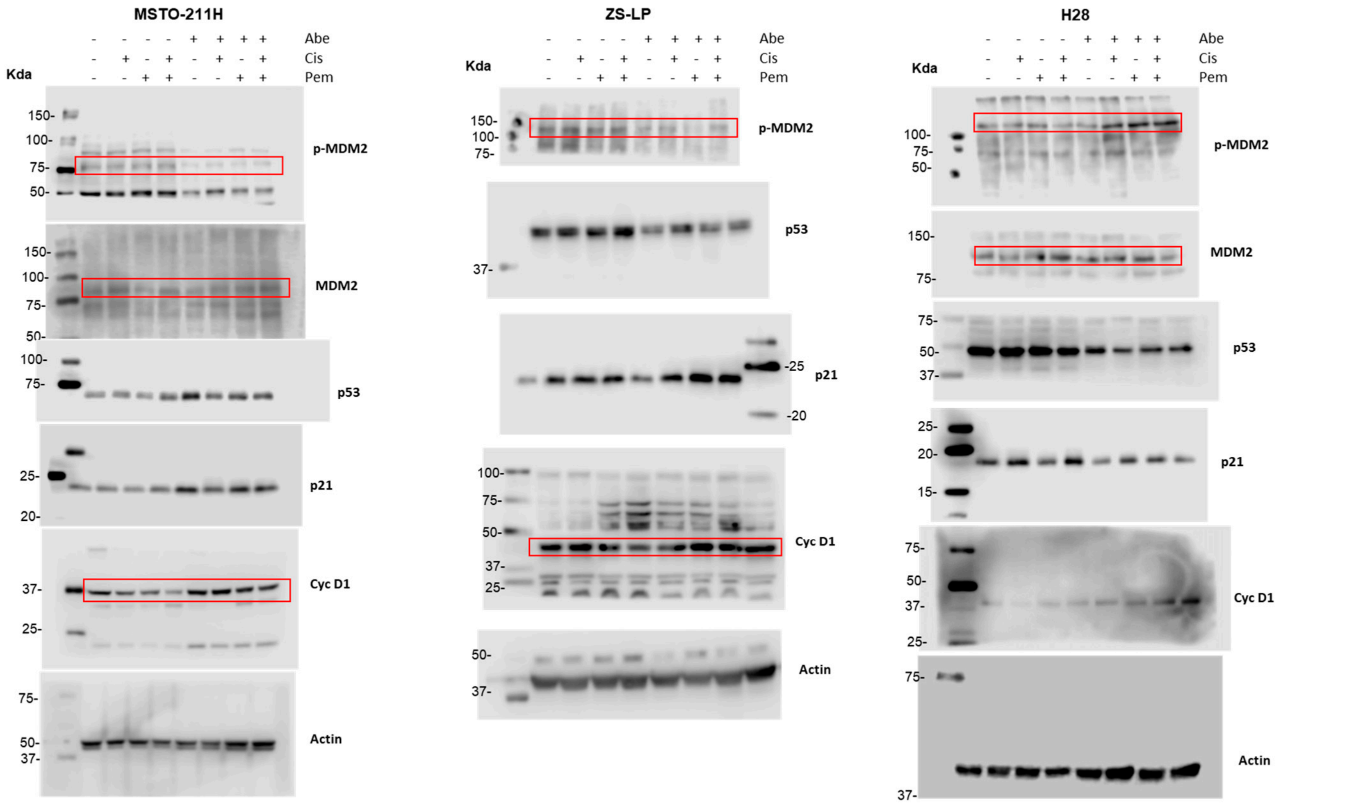

Figure S6: Full original blots used for Figure 4. Each blot membrane was cut based on the standard band positions and then incubated with the corresponding antibodies.

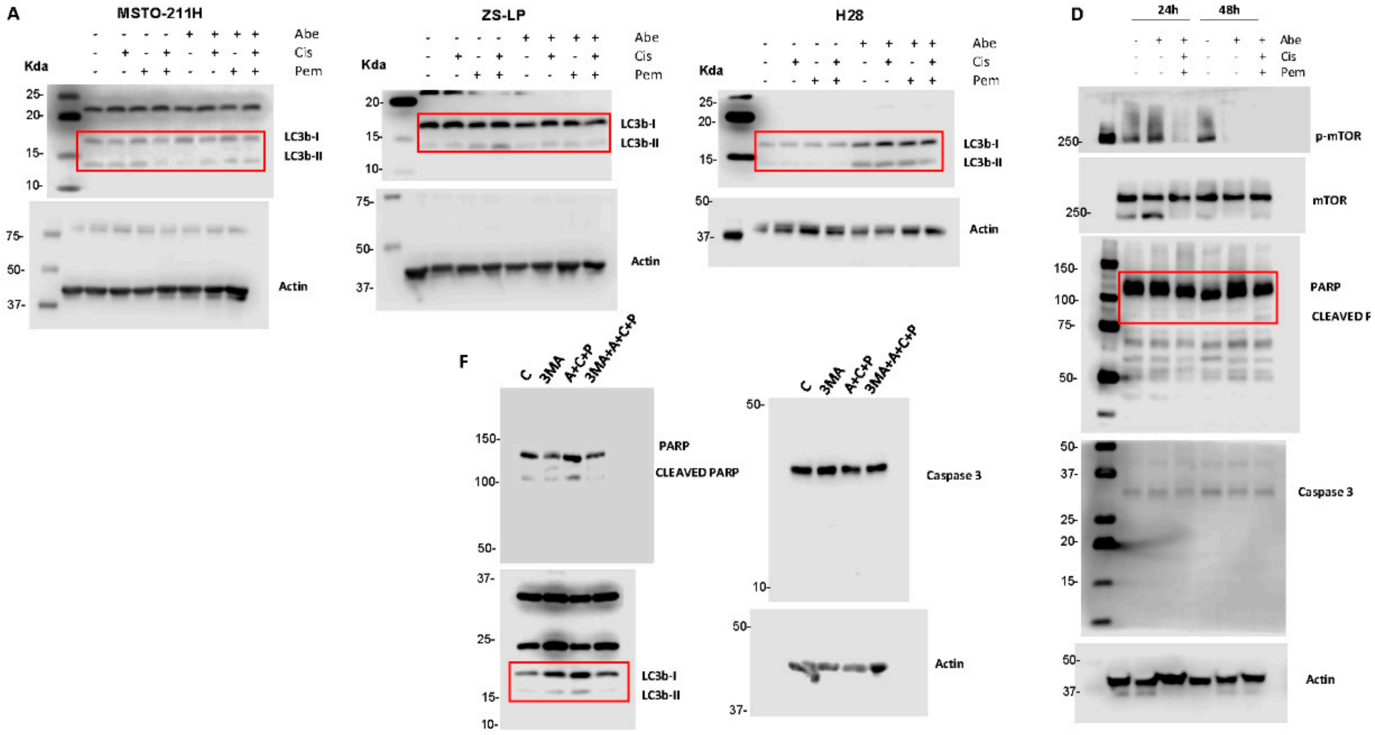

Figure S7: Full original blots used for Figure 5. Each blot membrane was cut based on the standard band positions and then incubated with the corresponding antibodies.
